# Pathologically diagnosed early‐stage gastric adenocarcinoma with enteroblastic differentiation after endoscopic submucosal dissection: A case report

**DOI:** 10.1002/deo2.318

**Published:** 2023-12-11

**Authors:** Hiroki Iwata, Hidehito Maeda, Kengo Tsuneyoshi, Takuma Matsumoto, Hiroshi Fujita, Yoshihiro Komohara, Akio Ido

**Affiliations:** ^1^ Digestive and Lifestyle Diseases Kagoshima University Graduate School of Medical and Dental Sciences Kagoshima Japan; ^2^ Department of Gastroenterology Izumi General Medical Center Kagoshima Japan; ^3^ Department of Cell Pathology, Graduate School of Medical Sciences Kumamoto University Kumamoto Japan

**Keywords:** alpha‐fetoprotein‐producing early‐stage gastric cancer, endoscopic finding, endoscopic submucosal dissection, gastric adenocarcinoma with enteroblastic differentiation, pathological finding

## Abstract

A 77‐year‐old male patient underwent esophagogastroduodenoscopy at his family doctor, and an easily hemorrhagic depressed lesion was noted near the anterior wall of the gastric antrum. A biopsy revealed moderately differentiated tubular adenocarcinoma > poorly differentiated adenocarcinoma, and the patient was referred to our department for further examination. A 15‐mm 0–IIc lesion is seen near the anterior wall of the gastric antrum and narrow band imaging magnifying endoscopy revealed obscured glandular duct structures and corkscrew pattern vascular structures. We diagnosed the patient with early‐stage gastric cancer [L, Ant, 15mm, cType0‐IIc, cT1(M‐SM1), cN0, cM0, cStage IA] after an esopahogastroduodenoscopy examination at our hospital, and endoscopic submucosal dissection was performed. Histopathological images with hematoxylin and eosin staining showed tumor cells with pale cytoplasm and the immunostaining for alpha‐fetoprotein, sal‐like protein 4, and Glypican3 was positive. The patient was pathologically diagnosed with gastric adenocarcinoma with enteroblastic differentiation, pT1b1 (SM, 0.4 mm), type 0–IIc, 15 mm, UL (‐), Ly0, and V0. Gastric adenocarcinoma with enteroblastic differentiation is one of the representative histological types of alpah‐fetoprotein‐producing gastric cancer. Alpha‐fetoprotein‐producing gastric cancer is infrequent, accounting for at least 3% of all gastric cancers, and is generally highly malignant. Most cases are already advanced upon diagnosis, and finding them in the early stage is rare. Therefore, pathological findings that may indicate the gastric adenocarcinoma with enteroblastic differentiation should be noted even in early gastric cancer.

## INTRODUCTION

Alpha‐fetoprotein (AFP)‐producing gastric cancer is infrequent and generally highly malignant. AFP‐producing gastric cancer is often not considered when treating lesions suspected of early‐stage gastric cancer because finding it in the early stage is rare. We report a case of early‐stage gastric cancer treated with endoscopic submucosal dissection (ESD), in which the pathological specimen revealed an AFP‐producing gastric cancer, especially one of its subtypes, gastric adenocarcinoma with enteroblastic differentiation (GAED).

## CASE REPORT

**FIGURE 1:**
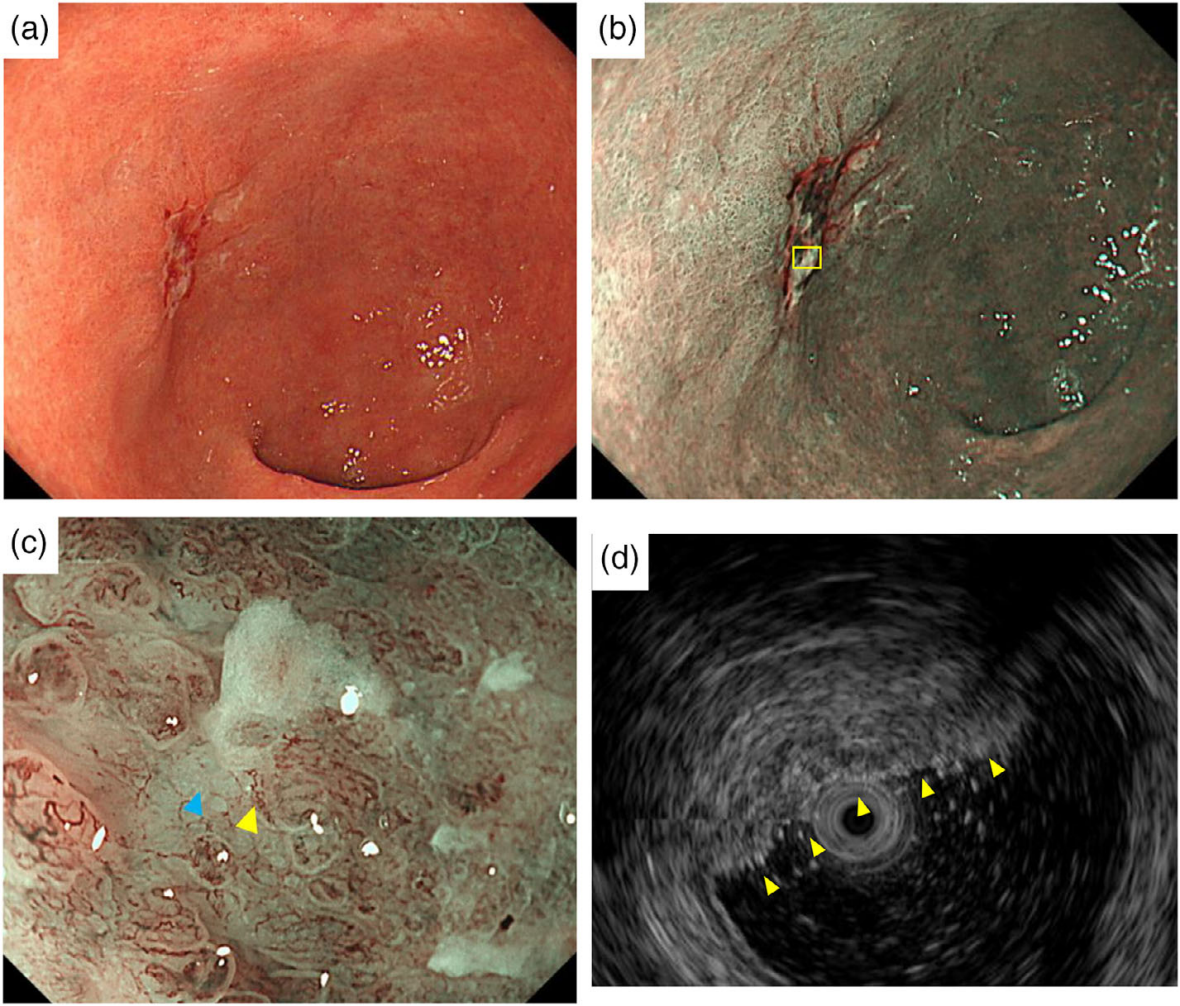
(a) A 15‐mm easily hemorrhagic 0–IIc lesion is seen near the anterior wall of the gastric antrum. The gastric wall progressed slightly well with air delivery. (b) Narrow band imaging . (c) Narrow band imaging magnifying endoscopy revealed partially obscured glandular duct structures (blue arrow) and corkscrew pattern vascular structures (yellow arrow). (d) Endoscopic ultrasonography revealed a hypoechoic mass, with no submucosal layer interruption.

**FIGURE 2:**
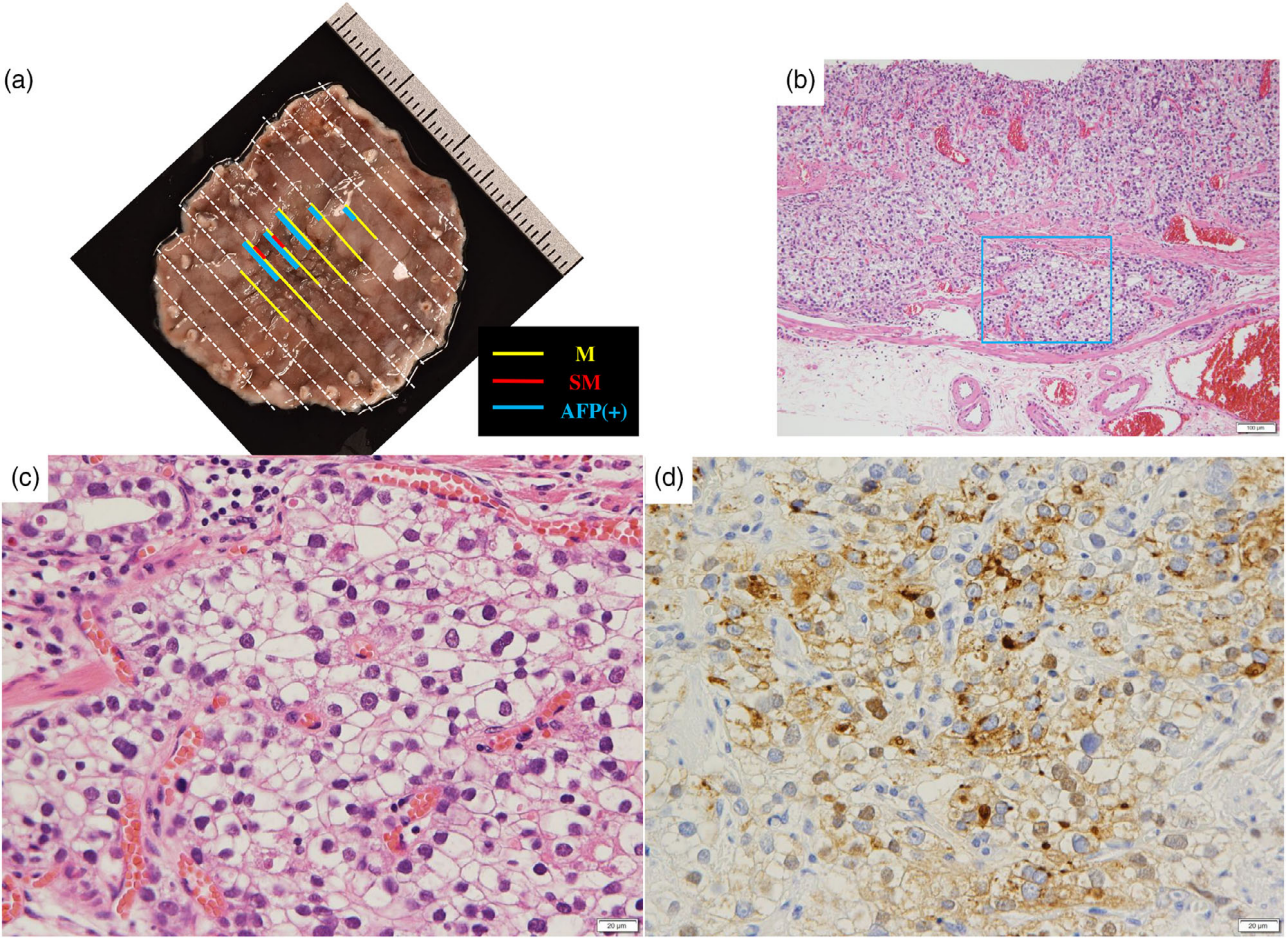
(a) The gross macroscopic image of the resected specimen, with yellow and red lines mapping tumor cells and blue lines mapping the alpha‐fetoprotein (AFP)‐producing site. (b) A microscopic pathological image with hematoxylin and eosin staining. (c) Tumor cells with pale cytoplasm were seen (blue box). (d) Immunostaining for AFP was positive.

**FIGURE 3:**
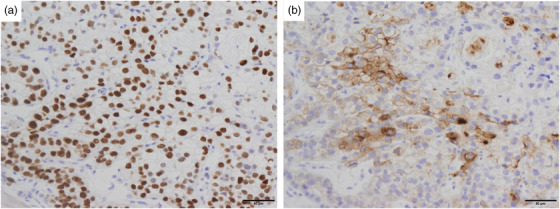
The immunostaining for sal‐like protein 4 (SALL4) (a) and Glypican3 (b) was positive.

A 77‐year‐old male patient underwent esophagogastroduodenoscopy at his family doctor in April 2022 to investigate his epigastric discomfort, and an easily hemorrhagic depressed lesion was noted near the anterior wall of the gastric antrum. Biopsy revealed a moderately differentiated tubular adenocarcinoma > poorly differentiated adenocarcinoma, and the patient was referred to our department for further examination. He has a height of 160.0 cm and a weight of 61.8 kg. Blood tests revealed no anemia. Tumor markers CEA and CA19‐9 were within normal range. *Helicobacter pylori* IgG antibody was positive at 10.3 IU/ml. Eosophagogastroduodenoscopy performed at our hospital revealed an atrophic background mucosa and a 15‐mm depressed lesion with redness near the anterior wall of the gastric antrum. The lesion was easily hemorrhagic with erosions within the depression. Gastric wall stretching by air delivery was slightly better (Figure 1a). Narrow band imaging magnifying endoscopy (NBI‐ME) revealed positive demarcation lines, irregular microvascular patterns, and irregular microsurface patterns according to the magnifying endoscopy simple diagnostic algorithm for gastric cancer.^1^ Further, NBI‐ME revealed partially obscured glandular duct structures and corkscrew pattern vascular structures (Figure 1b,c). Endoscopic ultrasonography revealed a hypoechoic mass, with no submucosal layer interruption (Figure 1d). Thoracic and abdominal contrast‐enhanced computed tomography (CT) scan revealed no obvious lymph node metastasis or liver metastasis but an irregularly shaped nodule of 13 mm in the apex of the left lung. The patient was scheduled for another chest CT scan 2 months after consultation with the respiratory surgery department. We diagnosed the patient with early‐stage gastric cancer [L, Ant, 15 mm, cType0‐IIc, cT1(M‐SM1), cN0, cM0, cStage IA] based on the above, and ESD was performed as a diagnostic treatment in May 2022. The lesion could be resected en bloc without complications by ESD. Figure 2a shows the gross macroscopic image of the resected specimen, with yellow and red lines mapping tumor cells. Figure 2b shows a microscopic pathological image with hematoxylin and eosin staining. It revealed tumor cells with pale cytoplasm (Figure 2c). The immunostaining for AFP was positive, and the patient was pathologically diagnosed with AFP‐producing gastric cancer (Figure 2d). The percentage of the AFP‐producing cell's component in the total tumor was 38.6%. In addition to AFP, the immunostaining of sal‐like protein 4 (SALL4) and Glypican3 was positive (Figure 3). Finally, the pathological diagnosis was GAED, pT1b1 (SM, 0.4 mm), type 0–IIc, 15 mm, UL (‐), Ly0, and V0. After the pathology results were obtained, the serum AFP level was measured and found to be within the normal range at 3.0 ng/ml. We reviewed the endoscopic images to ascertain whether we had diagnosed the GAED before ESD, however, NBI‐ME demonstrated obscured glandular duct structures and corkscrew pattern vascular structures, which is consistent with the poorly differentiated adenocarcinoma. The biopsy specimen with hematoxylin and eosin staining was also re‐examined; however, it was difficult to suspect GAED. The endoscopic curative grade was eCura C‐2, according to the Japanese Gastric Cancer Treatment Guidelines 2021,^2^ and additional surgical resection was decided. However, a CT scan in July 2022 revealed an enlarged nodule at the apex of the left lung and enlarged hilar and mediastinal lymph nodes on the same side, and confirmed the diagnosis of cStage IIIA left upper lobe lung cancer, and we decided to prioritize lung lesion treatment. Primary lung adenocarcinoma was diagnosed after an experimental resection and the specimen demonstrated negative AFP immunostaining. Currently, the patient continues to receive chemoradiotherapy followed by immune checkpoint inhibitors for lung cancer. No findings suggestive of recurrence have been detected 10 months after ESD.

## DISCUSSION

This case represents a novel finding, as it involved a detailed endoscopic observation, including NBI magnification, for GAED. However, GAED could not be diagnosed before ESD. GAED is one of the representative histological types of AFP‐producing gastric cancer. AFP‐producing gastric cancer is defined as gastric cancer with proven alpha‐fetoprotein production, which is infrequent, accounting for at least 3% of all gastric cancers. Generally, AFP‐producing gastric cancer is prone to liver metastasis, and most cases are already advanced upon diagnosis. Simultaneous liver metastasis demonstrated a detection rate of 30%, with a higher postoperative liver metastasis incidence than that of ordinary gastric cancer, and a shorter time until the metastasis appears.^3^ The blood AFP level correlates well with tumor growth. Histologically, it is characterized by large sporangia that are eosinophilic or pale, and AFP‐producing gastric carcinoma was suspected from hematoxylin and eosin staining of pathology in our case. The typical histological types of AFP‐producing gastric carcinoma are hepatoid adenocarcinoma (HAC), which is considered a rare/other histologic subtype of gastric adenocarcinoma, GAED, yolk sac tumor‐like carcinoma, and common types of adenocarcinoma that do not demonstrate the above histological features. They are classified by images of hematoxylin and eosin staining and immunostaining, and SALL4 and Glypican3 are known as sensitive markers for the diagnosis of not only AFPGC but also GAED.^4^, ^5^ In our case, the microscopic pathological image with hematoxylin and eosin staining revealed tumor cells with pale cytoplasm, and in addition to AFP, the immunostaining of SALL4 and Glypican3 was positive. For endoscopists, pathological specimen confirmation and discussion with a pathologist are important for diagnosing AFP‐producing early‐stage gastric cancer. AFP‐producing early‐stage gastric cancer was reported in 47 patients from 1990 to 2022, including our cases (Table 1).^3^, ^4^, ^5^, ^6^, ^7^, ^8^, ^9^, ^10^ Additionally, we could not find reports regarding early‐stage cases of AFP‐producing gastric cancer outside of Japan. The gross type was IIa+IIc/IIc+IIa in 23 cases (49%), and the histological types were por, tub2, and tub1, in that order. Most cases as high as 43 cases (91%) had SM carcinoma in depth. Liver metastasis was observed in 20 cases (43%), of which 5 cases (11%) had synchronous metastasis, despite early‐stage cancer. These results indicate that AFP‐producing gastric cancer, even in its early stage, may metastasize, and careful treatment strategy consideration and close follow‐up after treatment are necessary. Gokita et al. reported a case of AFP‐producing gastric cancer with lymph node recurrence despite the pathological diagnosis of curative resection after ESD.^10^ Therefore, even if the curability for AFP‐producing tumors shows eCura A after ESD, careful follow‐up is necessary.

**TABLE 1:** Reported cases of alpha‐fetoprotein (AFP)‐producing early‐stage gastric cancer.

Sex	M: 34 F: 13
Age	41–79 years
Macroscopic type	I: 4 IIa: 3 IIa + IIc: 23 IIc: 8 IIc + III: 1 unknown: 8
Histology	tub1: 9 tub2: 14 por: 15 pap: 2 HAC: 1 GAED: 2 HAC+GAED+CA: 2 unknown: 2
Size	3–65 mm
Depth	M: 4 SM: 43
Liver metastasis	Detected: 20 (synchronous 5) Not detected: 27

Abbreviations: CA, conventional adenocarcinoma; GAED, gastric adenocarcinoma with enteroblastic differentiation; HAC, hepatoid adenocarcinoma.

In conclusion, pathological findings that may indicate the GAED are necessary to be noted even in early‐stage gastric cancer.

## CONFLICT OF INTEREST STATEMENT

None.

## ETHICS STATEMENT

This study was conducted in accordance with the Declaration of Helsinki and with approval by the relevant institutional review board. The patient provided informed consent for the publication of this report.
